# Involvement of Large-Conductance Ca^2+^-Activated K^+^ Channels in Chloroquine-Induced Force Alterations in Pre-Contracted Airway Smooth Muscle

**DOI:** 10.1371/journal.pone.0121566

**Published:** 2015-03-30

**Authors:** Ming-Yu Wei, Lu Xue, Li Tan, Wen-Bo Sai, Xiao-Cao Liu, Qiu-Ju Jiang, Jinhua Shen, Yong-Bo Peng, Ping Zhao, Meng-Fei Yu, Weiwei Chen, Li-Qun Ma, Kui Zhai, Chunbin Zou, Donglin Guo, Gangjian Qin, Yun-Min Zheng, Yong-Xiao Wang, Guangju Ji, Qing-Hua Liu

**Affiliations:** 1 Institute for Medical Biology & Hubei Provincial Key Laboratory for Protection and Application of Special Plants in Wuling Area of China, College of Life Sciences, South-Central University for Nationalities, Wuhan 430074, China; 2 National Laboratory of Biomacromolecules, Institute of Biophysics, Chinese Academy of Sciences, Beijing 100101, China; 3 Department of Medicine, Acute Lung Injury Center of Excellence, University of Pittsburgh, Pittsburgh, PA 15213, United States of America; 4 Lankenau Institute for Medical Research & Main Line Health Heart Center, 100 Lancaster Avenue, Wynnewood, PA 19096, United States of America; 5 Department of Medicine-Cardiology, Feinberg Cardiovascular Research Institute, Northwestern University Feinberg School of Medicine, Chicago, IL 60611, United States of America; 6 Center for Cardiovascular Sciences, Albany Medical College, Albany, NY 12208, United States of America; Temple University School of Medicine, UNITED STATES

## Abstract

The participation of large-conductance Ca^2+^ activated K^+^ channels (BKs) in chloroquine (chloro)-induced relaxation of precontracted airway smooth muscle (ASM) is currently undefined. In this study we found that iberiotoxin (IbTx, a selective inhibitor of BKs) and chloro both completely blocked spontaneous transient outward currents (STOCs) in single mouse tracheal smooth muscle cells, which suggests that chloro might block BKs. We further found that chloro inhibited Ca^2+^ sparks and caffeine-induced global Ca^2+^ increases. Moreover, chloro can directly block single BK currents completely from the intracellular side and partially from the extracellular side. All these data indicate that the chloro-induced inhibition of STOCs is due to the blockade of chloro on both BKs and ryanodine receptors (RyRs). We also found that low concentrations of chloro resulted in additional contractions in tracheal rings that were precontracted by acetylcholine (ACH). Increases in chloro concentration reversed the contractile actions to relaxations. In the presence of IbTx or paxilline (pax), BK blockers, chloro-induced contractions were inhibited, although the high concentrations of chloro-induced relaxations were not affected. Taken together, our results indicate that chloro blocks BKs and RyRs, resulting in abolishment of STOCs and occurrence of contraction, the latter will counteract the relaxations induced by high concentrations of chloro.

## Introduction

It has been reported that chloro induced an increase in intracellular Ca^2+^ in ASM cells, however, which resulted in relaxation in precontracted ASM [1]. The relaxation was partially mediated by BKs [1, 2]. While, the BK-mediated relaxation was challenged by evidence that BK-mediated STOCs in ASM cells were completely blocked by chloro and that blockade of BKs failed to affect chloro-induced relaxation in precontracted ASM [3]. Recently, we [4] and others [5] have defined the mechanism underlying chloro-induced relaxation, which is due to inhibition of chloro on voltage-dependent L-type Ca^2+^ channels (VDCCs) and non-selective cation channels (NSCCs). However, the role of BKs in chloro-induced relaxation is still undefined. In this study, we found that chloro blocks RyRs and BKs, which results in STOC abolishment and contraction occurrence, the latter will counteract chloro-triggered relaxation. These results suggest that BKs are involved in chloro-induced relaxation in precontracted ASM.

## Materials and Methods

### Isolation of single ASM cells

Single mouse tracheal smooth muscle cells were enzymatically isolated as previously described [6]. Briefly, adult male BALB/c mice were euthanized by intraperitoneal injection of sodium pentobarbital (150 mg/kg) according to the protocol approved by the Institutional Animal Care and Use Committee of the South-Central University for Nationalities (Permit number: 2012-QHL-2). The tracheae were removed and transferred to an ice-cold solution containing 136 mM NaCl, 5.36 mM KCl, 0.44 mM KH_2_PO_4_SO_4_, 4.16 mM NaHCO_3_, 10 mM glucose, 10 mM HEPES, 0.34 mM NaHPO_4_·12H_2_O (pH 7.1, adjusted with NaOH). The epithelium, cartilage, and connective tissue were removed. The trachealis tissues were minced and incubated for 22 min at 35°C in the above solution supplemented with 2 mg/ml papain, 1 mg/ml dithioerythritol, and 1 mg/ml bovine serum albumin (BSA). The partially digested tissues were then transferred to the above solution supplemented with 1 mg/ml collagenase H, 0.15 mg/ml dithiothreitol, and 1 mg/ml BSA. After incubation for 8 min, the well-digested tissues were washed and gently triturated in the above solution to yield single smooth muscle cells. Cells were stored on ice and used for experiments within 4 h.

### Recordings of ion channel currents

Ion channel currents were measured using an EPC-10 patch-clamp amplifier (HEKA, Germany). BK-mediated STOCs were recorded using a classical whole-cell configuration [7]. Patch pipettes had a resistance of 3 to 5 MΩ when filled with an intracellular solution containing 74.5 mM KCl, 1 mM MgCl_2_, 10 mM HEPES, 64 mM K-aspartate, and 3 mM Na_2_ATP (pH 7.2, adjusted with KOH). The extracellular solution contained 130 mM NaCl, 5.5 mM KCl, 2.2 mM CaCl_2_, 1 mM MgCl_2_, 10 mM HEPES, and 5.6 mM glucose (pH 7.4, adjusted with NaOH). The holding potential was set at −40 mV. The junction potential, capacitance, and series resistance were compensated. The 30 s step voltage pulses were applied from −40 to +10 mV with an increment of 10 mV to record STOCs.

Single BK currents [8] were recorded at 0, 20, 40, and 60 mV using inside-out and outside-out patch clamp techniques under symmetrical K^+^ ion concentrations in the pipette and bath solutions. The intracellular solution contained 140 mM KCl, 1 mM MgCl_2_, 5 mM EGTA, 4.37 mM CaCl_2_ and 10 mM HEPES (pH 7.2, adjusted with KOH). The free Ca^2+^ ion concentration was 1 μM calculated using WEBMAXC STANDARD (http://www.stanford.edu/~cpatton/webmaxc/webmaxcS.htm). The extracellular solution contained 140 mM KCl, 1 mM MgCl_2_, 5 mM EGTA, 4.9 mM CaCl_2_ and 10 mM HEPES (pH 7.2, adjusted with KOH). The free Ca^2+^ ion concentration was 10 μM. Single channel currents were acquired at a digitization rate of 4 kHz and filtered at 1 kHz. Events were detected and all-point amplitude histogram and single channel open probability (*Po*) obtained using Clampfit 9 software (Axon Instruments; Foster, CA, USA) [9]. The histograms were fitted with the Gaussian distribution function. Peak values were obtained from these fitting traces and the net peak values were the amplitudes of single BK currents.

### Measurements of intracellular Ca^2+^

For the measurements of Ca^2+^ sparks, single mouse tracheal smooth muscle cells were loaded with 2.5 μmol/L fluo-4 AM. Ca^2+^ sparks were measured using an LSM 700 confocal scanning laser microscope (Carl Zeiss, Göttingen, Germany) and Zen 2010 software (Carl Zeiss, Göttingen, Germany). The frequency (sparks/s/μm) and amplitude of Ca^2+^ sparks were analyzed using Zen 2010 software and Interactive Data Language software (IDL, Research Systems, Boulder, CO, USA) [10]. Simultaneous recordings of Ca^2+^ sparks and STOCs were performed by a method that combined a confocal scanning laser microscope with the patch-clamp amplifier [6].

The global Ca^2+^ changes were measured using confocal microscope as described previously [4], which were also measured using fura-2 AM dye. For the latter measurement, single mouse ASM cells were loaded with 4 μmol/L fura-2AM. Fluorescence images were acquired at 340 nm and 380 nm and analyzed using the TILL Polychrome V monochromator system (Chroma Technology, Brattleboro, VT, USA) and Metafluor for Olympus software (TILL, Gräfelfing, Germany). The ratio of fluorescence intensity at 340 nm and 380 nm was calculated and used as the index for the intracellular Ca^2+^ level [11].

### Measurement of ASM tension

ASM tension was measured in mouse tracheal rings as previously reported [4]. In brief, tracheae were obtained and quickly transferred into an ice-cold solution (135 mM NaCl, 5 mM KCl, 1 mM MgCl_2_·6H_2_O, 2 mM CaCl_2_, 10 mM HEPES, 10 mM glucose, pH 7.4) equilibrated with 5% CO_2_/95% O_2_. Connective tissue was removed and four cartilage tracheal rings were cut from the same distal tracheae. Each ring was mounted horizontally in a 10 ml organ bath chamber containing the above solution equilibrated with 5% CO_2_/95% O_2_. The preload was 0.3 g. During a 60-min equilibration period, the rings were washed every 15 min and then precontracted twice with ACH (10^−4^ M). After the rings rested for an additional 30 min, experimental measurements of tension were carried out.

### Reagents

Papain, dithioerythritol, dithiothreitol, collagenase H, bovine serum albumin, 2-aminoethoxydiphenyl borate (2-APB), acetylcholine (ACH) and chloroquine (chloro) were purchased from Sigma. Iberiotoxin (IbTx), paxilline (pax), ryanodine and U73122 were purchased from Cayman Chemical. Fluo-4 AM and fura-2 AM were from Molecular Probes.

### Data analysis

Results are expressed as means ± SEM. Comparisons between two groups were performed with the Student’s *t*-test using Origin 9.0 software (OriginLab, Northampton, MA, USA), and *p* < 0.05 was considered statistically significant.

## Results

### Chloro blocks BK-mediated STOCs

**Fig 1:**
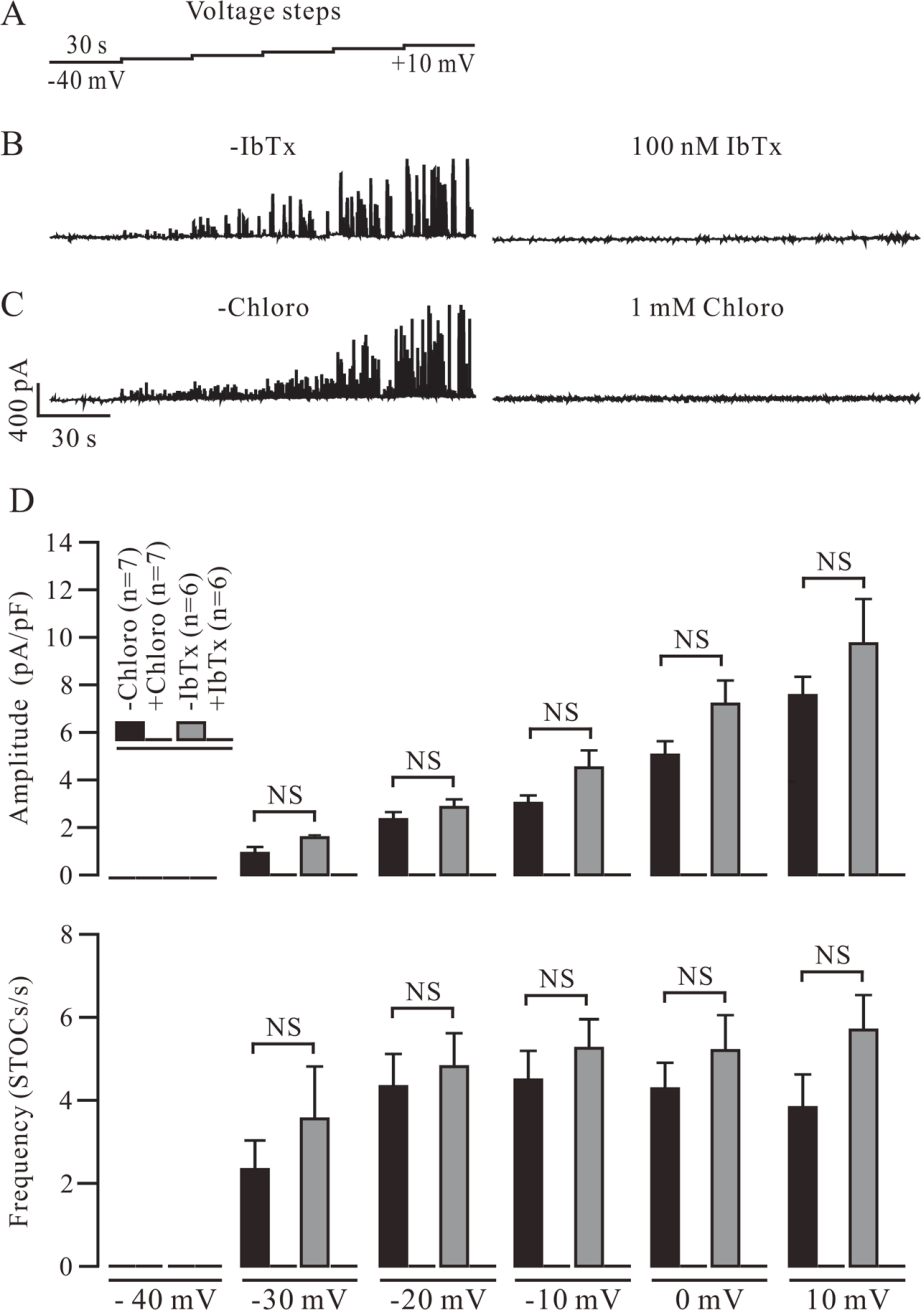
Chloroquine (chloro) blocks BK-mediated STOCs. (A) Mouse tracheal smooth muscle cells were patched using a whole-cell configuration and held at −40 mV. STOCs were recorded using a series of 30 s voltage steps from −40 through +10 mV. (B, C) Following the addition of the selective BK blocker iberiotoxin (IbTx, 100 nM, in 6 cells) or chloro (1 mM, in 7 cells), STOCs were abolished. (D) The averaged amplitude and frequency of STOCs in the absence and presence of IbTx. NS (not significant) denotes *p* > 0.05. These data indicate that chloro blocks BK-mediated STOCs.

To study the role of BKs in chloro-induced relaxation, we first sought to determine whether these channels can be blocked by chloro. The voltage steps shown in Fig. 1A were used to record BK-mediated STOCs in single mouse tracheal smooth muscle cells using the patch clamp technique [12]. The STOCs were abolished by IbTx (100 nM), a selective blocker of BKs (Fig. 1B), which indicates that the STOCs were BK currents. We then observed the effect of chloro on the currents and found that they were completely blocked by 1 mM chloro (Fig. 1C). The averaged amplitude and frequency of the STOCs in the absence and presence of IbTx or chloro are shown in Fig. 1D. Together these results suggest that chloro blocks BK-mediated STOCs.

We next assessed the dose-dependent inhibition of chloro on the STOCs. The STOCs were recorded at 10 mV, which were gradually inhibited following cumulative additions of chloro (Part A of S1 Fig.). The amplitude and frequency of the STOCs from 5 cells are summarized (Part B of S1 Fig.). These results are in agreement with the previous findings that STOCs will be blocked by chloro [3]. However, on the basis of these results, we cannot conclude that the disappearance of STOCs was only due to the direct blockage of BKs by chloro, because the blockade of RyRs can also result in abolishment of STOCs.

### Chloro blocks RyRs

**Fig 2:**
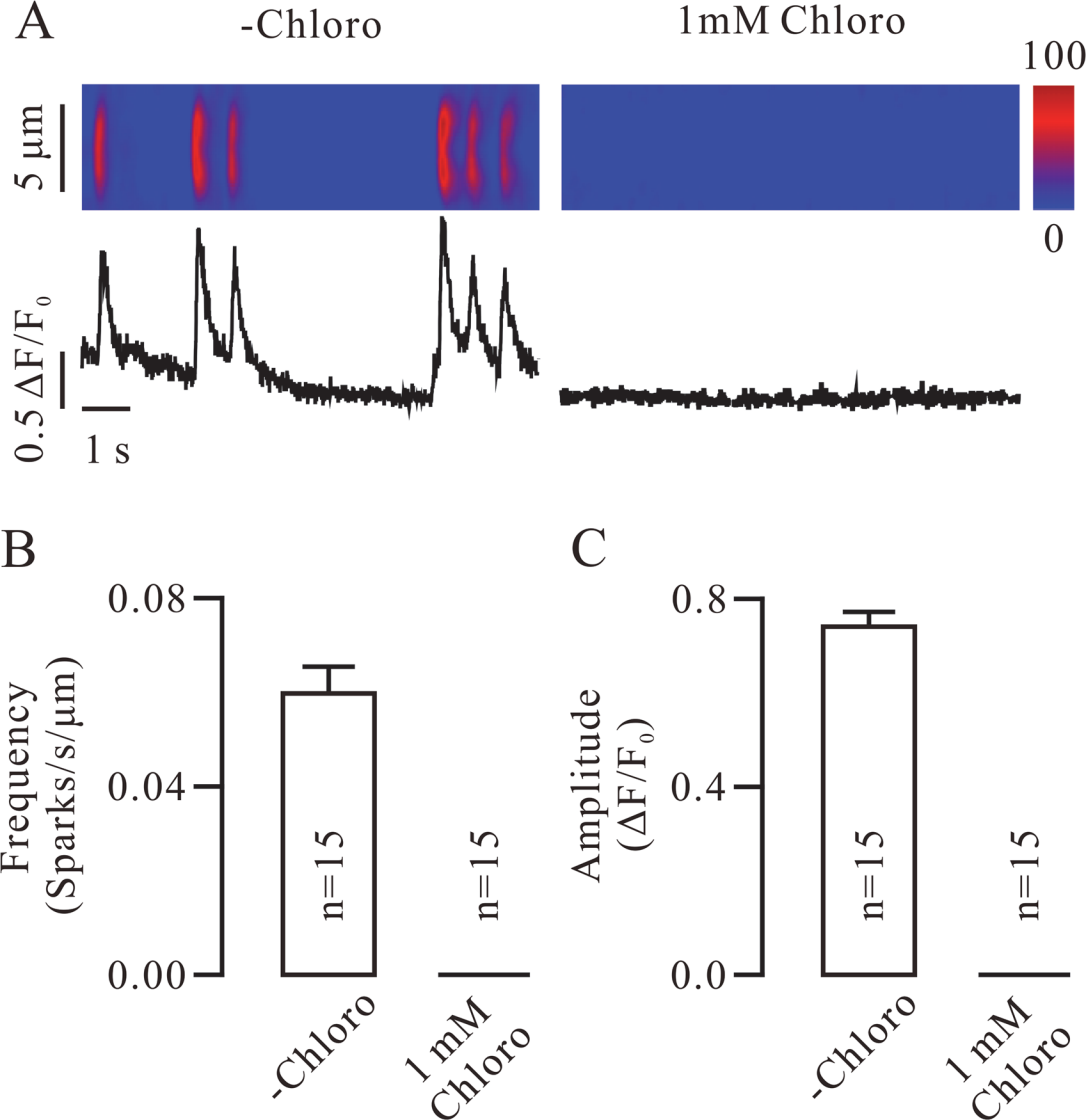
Chloro blocks Ca^2+^ sparks. (A) Ca^2+^ sparks in a single tracheal smooth muscle cell (*left*) were abolished by 1 mM chloro (*right*). (B, C) The frequency and amplitude of Ca^2+^ sparks from 15 cells. These results indicate that chloro inhibits RyRs.

**Fig 3:**
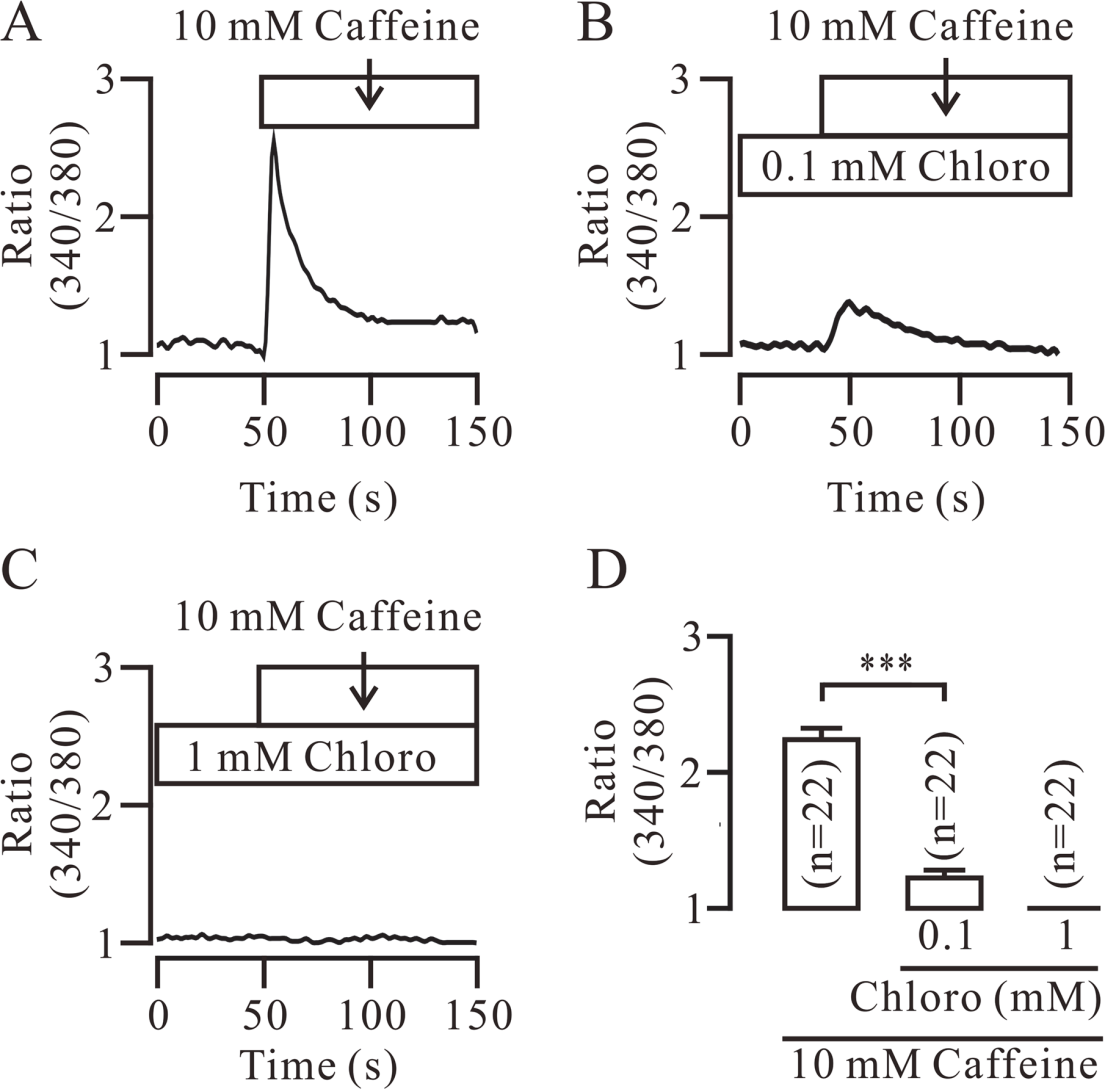
Chloro blocks caffeine-induced Ca^2+^ increases. (A) The selective RyR activator caffeine (10 mM) induced an increase in intracellular Ca^2+^ that was significantly and completely inhibited by 0.1 mM (B) and 1 mM chloro (C), respectively. Each experiment was conducted in 22 cells and the summarized results are shown in (D). *** denotes *p* < 0.001. These results further support that chloro inhibits RyRs.

We next examined whether the RyRs will be inhibited by chloro via observing its effect on RyR-mediated Ca^2+^ sparks. We found that Ca^2+^ sparks were markedly inhibited and abolished by 0.1 mM (S2 Fig.) and 1 mM chloro (Fig. 2), respectively. These results indicate that chloro can inhibit RyRs. In order to further confirm it, we used caffeine (10 mM), a selective activator of RyRs, to stimulate RyRs inducing a cytosolic Ca^2+^ increase and observed whether it will be affected by chloro. Caffeine induced a Ca^2+^ increase, which was significantly and completely inhibited by 0.1 and 1 mM chloro, respectively (Fig. 3), indicating that chloro is able to completely block caffeine-induced Ca^2+^ elevation, further implying chloro inhibits RyRs. Since chloro was previously shown to induce Ca^2+^ increases [1, 5], we thus evaluated the effect of chloro on intracellular Ca^2+^ and found that chloro (0.1 mM) induced a small increase in Ca^2+^ and inhibited the following 10 mM caffeine-induced Ca^2+^ increases (S3 Fig.). These results suggest that chloro not only induces Ca^2+^ increases but also inhibits the caffeine-induced Ca^2+^ increases.

Although caffeine-induced Ca^2+^ increases were inhibited by chloro, caffeine is a weak bitter tastant, which would also be able to induce Ca^2+^ elevations via type 2 taste receptors (T2Rs). Thus, we observed the effect of 2-APB, an inhibitor of inositol 1,4,5-triphosphate receptor (IP_3_Rs), on the caffeine-induced Ca^2+^ elevations and found that 2-APB significantly inhibited the increases in Ca^2+^ triggered by 10 mM caffeine (S4 Fig.), suggesting that caffeine-induced Ca^2+^ elevations are partially mediated by IP_3_Rs. We replaced 2-APB with the phospholipase C (PLC) inhibitor U73122 (1 μM) and repeated the same experiments and found that U73122 also inhibited caffeine-induced Ca^2+^ increases (S5 Fig.). This result indicates that PLC mediates caffeine-induced Ca^2+^ elevations. All these data imply that caffeine will activate T2Rs to induce Ca^2+^ elevations via G protein-PLC-IP_3_-IP_3_R pathway. To further confirm this conclusion, we did the following experiments. We used 10 mM caffeine to open the RyRs and 30 μM ryanodine to block them. After washing both out, we observed whether caffeine can induce Ca^2+^ increases. The result showed that caffeine still triggered Ca^2+^ elevations (S6 Fig.), indicating that caffeine will employ RyR-independent pathway to induce Ca^2+^ increases, supporting above finding that caffeine can induce Ca^2+^ increases through T2Rs-G protein-PLC-IP_3_-IP_3_R pathway.

**Fig 4:**
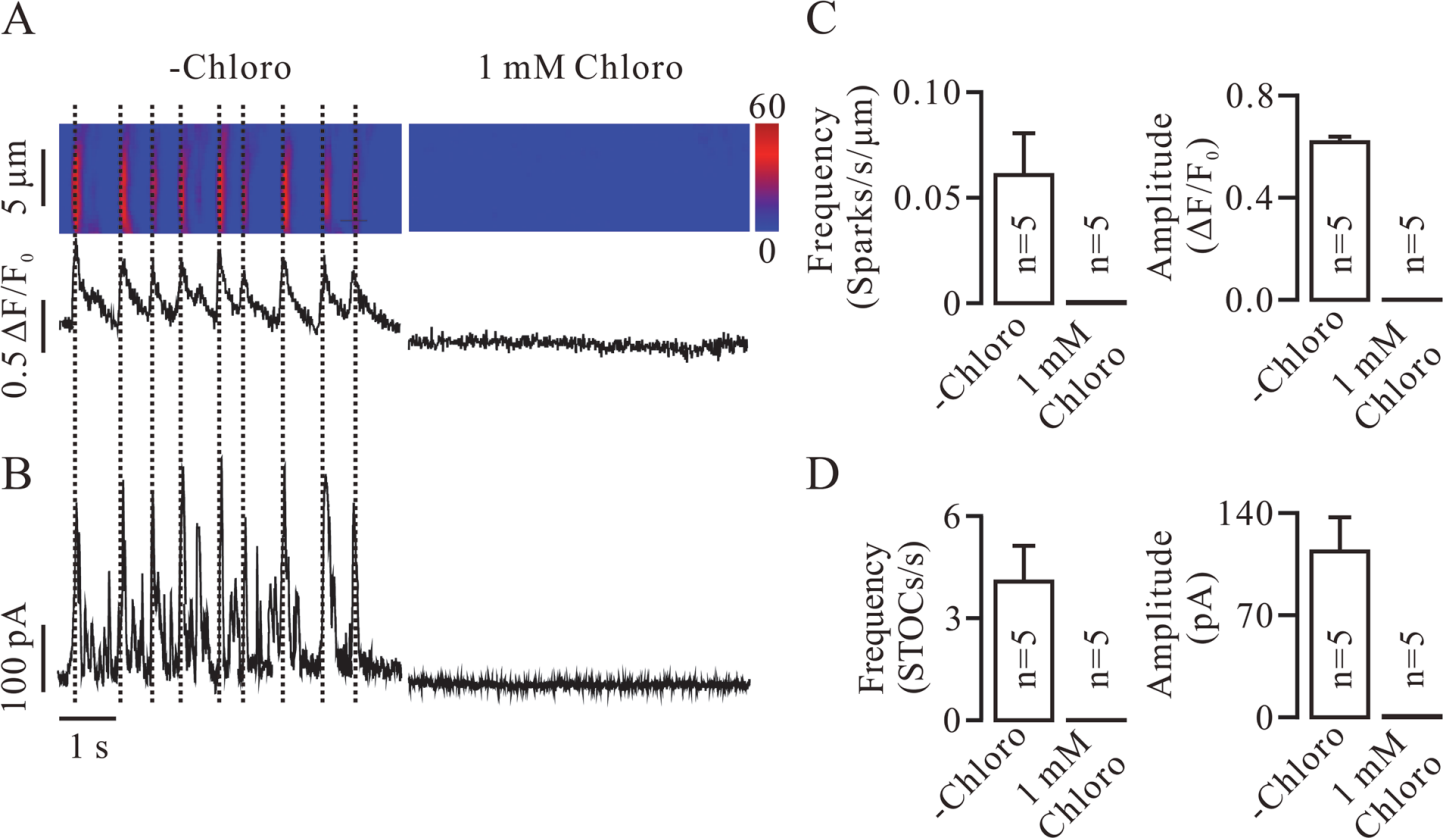
Chloro simultaneously inhibits Ca^2+^ sparks and STOCs. (A, B) A mouse tracheal smooth muscle cell was patched and held at −40 mV. Ca^2+^ sparks and STOCs were simultaneously recorded at 0 mV, which were blocked after adding 1 mM chloro. Ca^2+^ spark-triggered STOCs are indicated by the dashed lines. (C, D) The frequency and amplitude of Ca^2+^ sparks and STOCs from 5 cells are summarized. These data further demonstrate that the disappearance of STOCs will be due to the blockade of RyRs by chloro.

To further observe the inhibition of chloro on RyRs and BKs, we simultaneously recorded Ca^2+^ sparks and STOCs in single tracheal smooth muscle cells. Cells were patched and held at −40 mV, and Ca^2+^ sparks and STOCs were then measured. Ca^2+^ spark-triggered STOCs were observed (*left* of Fig. 4A and B). Following the addition of 1 mM chloro, both were abolished (*right* of Fig. 4A and B). The mean frequencies and amplitudes of the Ca^2+^ sparks and STOCs were calculated (Fig. 4C and D). These data demonstrate that the abolishment of BK-mediated STOCs was due to inhibition of chloro on RyRs.

### Chloro blocks BKs

**Fig 5:**
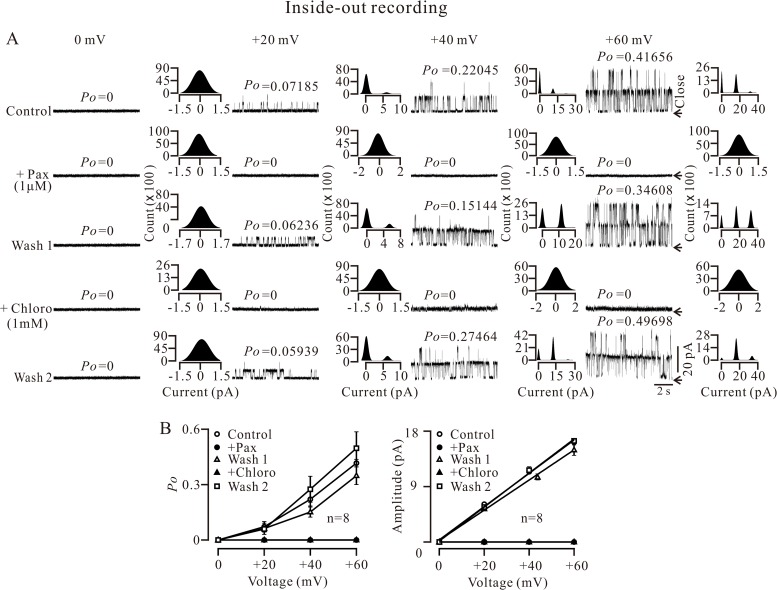
Intracellular chloro blocks single BK currents. (A) A cell was patched and an inside-out configuration was formed. The holding potential was 0 mV. Single channel currents were then recorded at 0, 20, 40, and 60 mV, which were blocked by 1μM paxilline (pax, another selective blocker of BKs), indicating that these currents were BK currents. After pax washout, the currents resumed and were abolished by 1 mM chloro (from the intracellular side). After washing out the chloro, the currents re-occurred. The closed state is indicated by arrows. The *Po* values were calculated and presented above the traces. All-point histograms were constructed and fitted with the Gaussian distribution function. The amplitudes of single channel currents were then obtained. (B) Based on the values from 8 experiments, the relationships between the *Po* and voltage, and amplitude and voltage were plotted. These data indicate that chloro blocks BKs completely from the intracellular side.

Although our data have shown chloro inhibited BK-mediated STOCs, we did not know whether chloro directly blocks BKs. To clarify this, we measured single BK currents using an inside-out configuration with single channel recording. The holding potential was 0 mV and single channel currents were recorded from an excised patch at 0, 20, 40 and 60 mV. The currents were abrogated following the addition of 1 μM pax, another selective blocker of BKs [13, 14] (Fig. 5A), confirming that these single channel currents are BK currents. After pax washout, the currents were restored and then subsequently completely and reversibly blocked by the addition of 1 mM chloro. The open probability (*Po*) values were calculated and presented above each trace (Fig. 5A). All-point amplitude histograms were also constructed, from which the amplitudes of single BK currents can be obtained. The *Po*-voltage and current-voltage curves were plotted based on the values acquired from experiments conducted on 8 excised patches from 8 cells (Fig. 5B). These results indicate that chloro can directly block BKs from the intracellular side.

**Fig 6:**
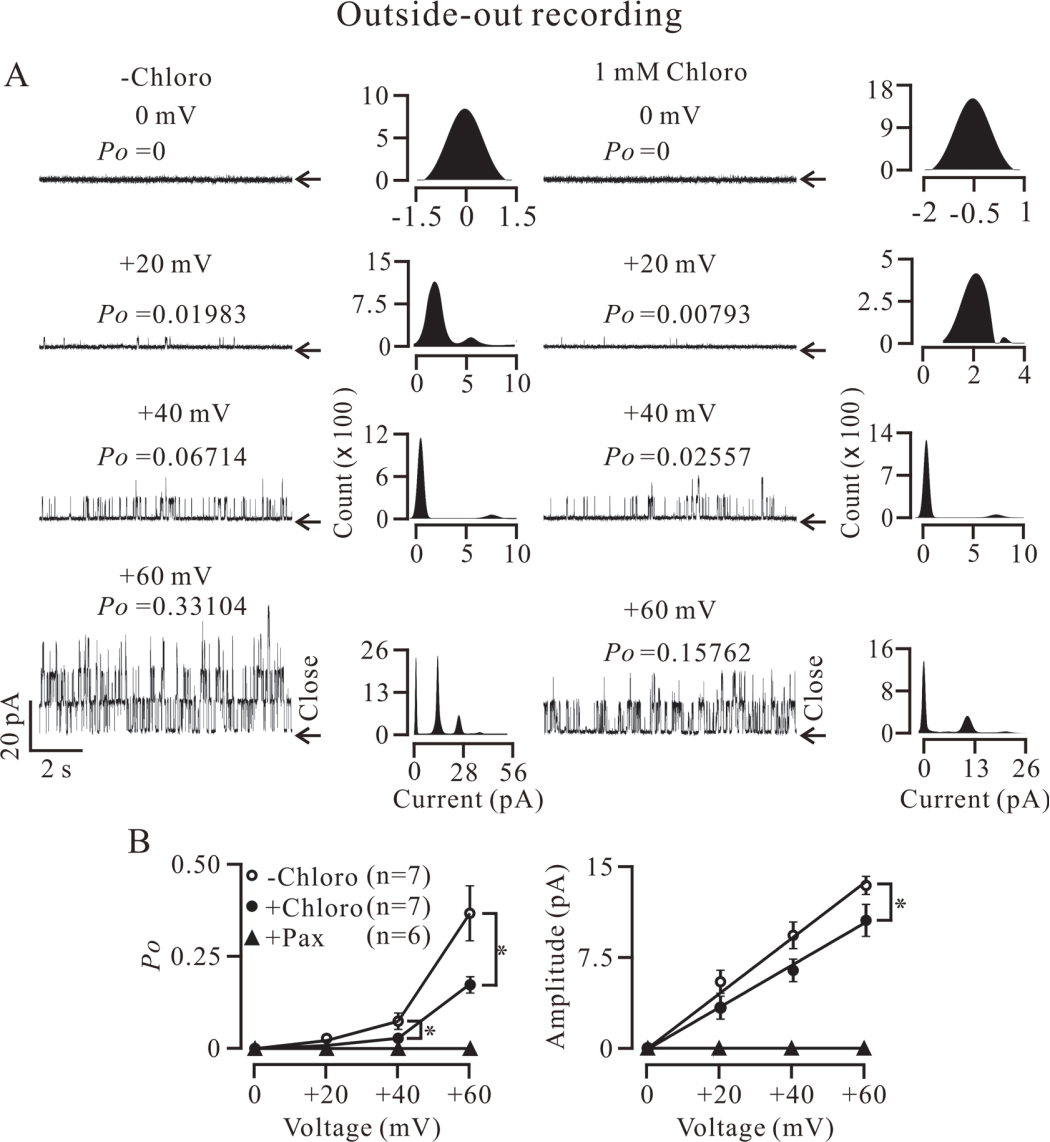
Extracellular chloro inhibits single BK currents. (A) Single channel currents were recorded using an outside-out approach. Following the addition of 1 mM chloro (from the extracellular side), single channel activities were partially inhibited. The *Po* values and amplitudes of single channel currents were calculated as described in Fig. 5. (B) The curves from 7 experiments show that the mean *Po* (at 40 and 60 mV) and amplitude (at 60 mV) were decreased. * denotes *p* < 0.05. These single channel currents were completely blocked by pax (n = 6), indicating these currents are BK currents. These results demonstrate that chloro partially inhibits BKs from the extracellular side.

Single BK currents were also recorded with an outside-out configuration, and the currents were inhibited following the application of 1 mM chloro (Fig. 6A and B). The *Po* (at 40 and 60 mV) and current amplitude (at 60 mV) were significantly inhibited in the presence of chloro (Fig. 6B). As an additional control, we evaluated the effect of pax on the currents and found that pax completely blocked the single channel currents from the extracellular side (Fig. 6B), indicating that the single currents were BK currents. These results demonstrate that chloro can partially inhibit BKs from the extracellular side.

### Effect of chloro on ASM force

**Fig 7:**
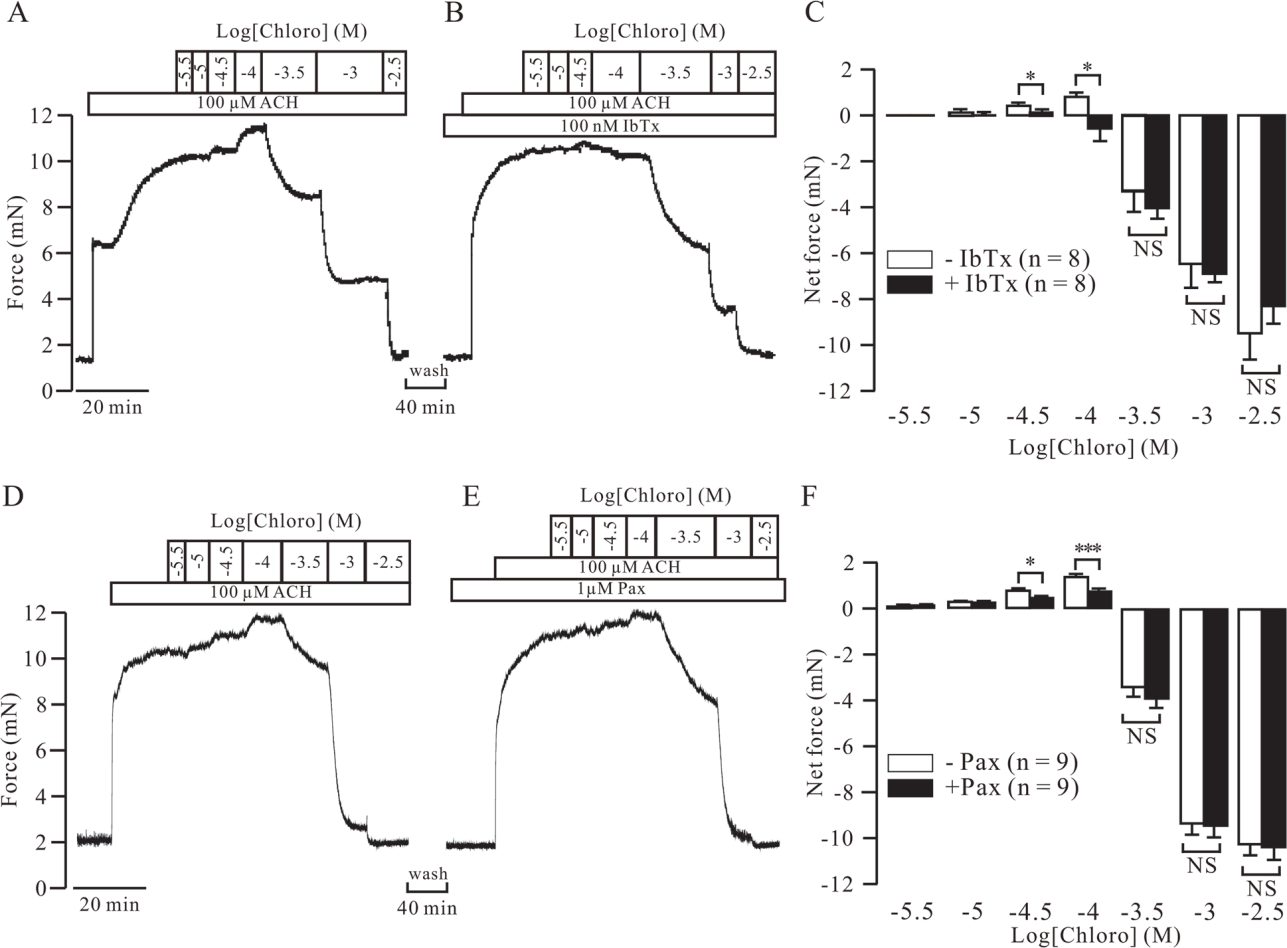
Role of BKs in chloro-induced force changes in precontracted mouse ASM. (A) ACH induced a steady state contraction in a mouse tracheal ring. Following cumulative additions of chloro, additional contractions were observed prior to the relaxations. (B) The same ring was washed and allowed to rest for 40 min. IbTx was then added and the same experiments were performed. The chloro-induced contractions were inhibited, the relaxations were not affected. (C) Averaged net changes in force from 8 experiments. In the absence of IbTx, lower concentrations (31.6 and 100 μM) of chloro induced contractions compared to that in the presence of IbTx. However, the relaxations in the two groups showed no differences. (D, E, F) The identical experiments were performed except that IbTx replaced by pax and similar results were obtained from 9 experiments. * denotes *p* < 0.05; *** denotes *p* < 0.001; NS denotes *p* > 0.05. To calculate the net contractile forces, the ACH-induced peak value was as the reference level, while the total value of ACH- and blocker-induced peak value will be the reference level for relaxant calculation. These data indicate that BKs involve in chloro-induced force alterations.

Based on above results, BKs would be involved in chloro-induced relaxation of precontracted ASM because activation of these channels can result in relaxation [15–19]. To test this hypothesis, we measured the changes in force following cumulative additions of chloro in ACH-precontracted mouse tracheal rings in the absence and presence of 100 nM IbTx and 1 μM pax (the concentration that completely blocked BKs as shown in Figs. 1, 5 and 6). After the ACH-induced contraction reached a plateau, low concentrations of chloro (31.6 and 100 μM) evoked significant additional contractions, although from 0.316 mM and 1 through 3.16 mM, chloro induced relaxations to baseline level (Fig. 7A and D). These results indicate that the blockade of BKs by chloro induced contraction was reversed into relaxation following increases of chloro concentration. After washing and then resting for 40 min, 100 nM IbTx or 1 μM pax was added and the same experiments were conducted (Fig. 7B and E). Following blockage of BKs by IbTx or Pax, chloro-induced contractions were inhibited, while chloro-induced relaxations were unaffected relative to that in the absence of IbTx and pax (Fig. 7C and F). These data further support that chloro-induced contraction was due to its blockade on BKs. Such contraction will counteract high concentrations of chloro-induced relaxations. Thus, BKs involve in chloro-induced relaxation.

In time control experiments the biphasic effect of chloro (Parts A and C of S7 Fig.) was not altered after 40 min (Parts B and C of S7 Fig.), indicating that the disappearance of contractions was not due to the 40 min resting time. In order to further demonstrate that BKs are involved in the force changes, we observed the effect of pax on the force and found it resulted in a contraction (S8 Fig.), confirming that blockade of BKs can induce contraction.

## Discussion

Our data demonstrate that chloro blocks both BKs and RyRs inducing STOC abolishment and contraction occurrence. The contraction will counteract high concentration of chloro-induced relaxation of precontracted ASM.

In the present study, we investigated the effect of BKs on chloro-induced relaxation in precontracted ASM. Two studies have shown that BKs mediated chloro-induced relaxation in precontracted ASM [1, 2]. Meanwhile, another work showed chloro blocked BK-mediated STOCs and BK blockers had no effect on chloro-induced relaxation in precontracted ASM, suggesting that BKs are not involved in chloro-induced relaxation [3]. To resolve this apparent inconsistency, we first needed to know whether chloro can block BKs. We recorded Ca^2+^ spark-activated BK currents (STOCs) and found that they were completely blocked by a specific blocker of BKs, IbTx (Fig. 1B), suggesting that these STOCs are BK-mediated currents. Moreover, the currents can also be inhibited by chloro in dose-dependent manner (Fig. 1C; S1 Fig.), in agreement with the previous findings that chloro inhibited STOCs [3].

The inhibition of STOCs can be due to the inhibition of RyRs and/or BKs by chloro. In fact, chloro inhibited Ca^2+^ sparks (Fig. 2; S2 Fig.) and caffeine-induced Ca^2+^ increases (Fig. 3; S3 Fig.), indicating that chloro blocks RyRs. This will be further supported by dual recording experiments of Ca^2+^ sparks and STOCs (Fig. 4). Thus, inhibition of chloro on RyRs will be one reason for the disappearance of STOCs. To further clarify whether the inhibition of STOCs was due to that chloro directly blocks BKs, we recorded single BK currents in inside-out and outside-out excised patches. Chloro directly and completely blocked BKs from the intracellular side and partially from the extracellular side (Figs. 5 and 6). These data indicate that chloro can block RyRs and BKs, resulting in STOCs to disappear. This implies that BKs would be involved in chloro-induced relaxation of precontracted ASM.

In the next, we further observed the effect of BK blockers, IbTx and pax, on the force (Fig. 7). In the absence of blockers, chloro induced additional contractions and then relaxations. This indicates that the contractions will result from the blockade of BKs by chloro, however, the relaxations would be due to the blockade of both VDCCs and NSCCs by chloro [4, 5]. To further confirm this conclusion, the same experiments were carried out following blockade of BKs with IbTx of Pax. Under this condition, chloro-induced contractions were inhibited (Fig. 7), which will not be due to 40 min waiting time (S7 Fig.), confirming that the chloro-caused contractions were due to its blockade on BKs. The contraction induced by chloro will then counteract the following relaxations evoked by high concentrations of chloro. Thus, BKs will involve in chloro-induced relaxation. To further reveal the role of BKs, we observed the force changes following the addition of pax and found that it induced a contraction (S8 Fig.), supporting the results that blockade of BKs by chloro will induce contraction (Fig. 7).

In this study, caffeine was used to test whether RyRs were inhibited (Fig. 3; S3 Fig.). In fact, caffeine is a weak bitter tastant, thus, a question will be raised whether caffeine can stimulate T2Rs to induce Ca^2+^ increases. Our experiments demonstrated that caffeine-triggered Ca^2+^ increases will be partially mediated by T2Rs because following RyR open by caffeine and blockade by ryanodine, caffeine still induced Ca^2+^ increases (S6 Fig.), suggesting caffeine might cause Ca^2+^ elevations via T2Rs. This was further supported by that the inhibition of IP_3_R-PLC (S4 and S5 Figs.), the downstream signal pathway of T2Rs, resulted in decreases in caffeine-induced Ca^2+^ increases. However, why caffeine failed to evoke responses in the presence of 1 mM chloro (Fig. 3), the reason would be because that chloro inhibited both IP_3_Rs [20] and RyRs, resulting in caffeine cannot induce Ca^2+^ increases.

Although the inhibition of chloro on RyRs has been demonstrated in this study, which is not consistent with the previous results showing that chloro (0.1 mM) failed to inhibit an increase in Ca^2+^ induced by 20 mM caffeine [20]. This discrepancy may have occurred because: (1) the diffusion of chloro into ASM cells in lung slices was limited compared to single cells, or (2) the concentration of chloro was not high enough (we used the higher concentration of 1 mM).

Based on above interpretations, chloro can inhibit RyRs and IP_3_Rs abolishing caffeine-induced Ca^2+^increases, however, why chloro induced a Ca^2+^ elevation (S3 Fig.). The reason would be because that chloro can not only activate T2R-G protein-PLC-IP_3_-IP_3_R pathway to induce Ca^2+^ increases [1, 5], but also inhibit IP_3_Rs to decrease Ca^2+^ that supported by our results that chloro blocked caffeine-induced Ca^2+^ increases partially via IP_3_Rs (Fig. 3; S4 Fig.). Thus, whether the chloro-induced Ca^2+^ elevations occur or not would be determined by the degree of chloro-mediated direct inhibition of IP_3_Rs and activation of IP_3_Rs via T2R-G protein-PLC-IP_3_-IP_3_R pathway. That degree will be related to chloro concentration. In this study, 0.1 mM chloro induced Ca^2+^ increases. However, the detailed mechanism needs to be further investigated.

In summary, our data demonstrate that chloro blocks RyRs and BKs (completely from the intracellular side and partially from the extracellular side) to abolish STOCs. The blockade of BKs induces a contractive component that decreases but does not prevent chloro-induced complete relaxation of precontracted ASM. These findings imply that BKs involve in chloro-induced ASM relaxation and that the activators of BKs and chloro might be the potential bronchodilators.

## Supporting Information

S1 Fig Chloro inhibits STOCs. (A) STOCs were recorded at 10 mV, which were gradually inhibited by chloro. (B) The dose-dependent inhibition of amplitude and frequency from 5 cells. * denotes *p* < 0.05 (versus control); ** denotes *p* < 0.01 (versus control). These data show that chloro dose-dependently inhibits STOCs.
(PDF)

S2 Fig Chloro inhibits Ca^2+^ sparks. Ca^2+^ sparks from 8 cells were measured as described in Fig. 2A. (A) The frequency and (B) amplitude were markedly inhibited by 0.1 mM chloro. * denotes *p* < 0.05; *** denotes *p* < 0.001. These experiments demonstrate that low concentrations of chloro do not completely block, but rather inhibit RyRs.
(PDF)

S3 Fig Effect of chloro and caffeine on intracellular Ca^2+^ levels. (A, B) Caffeine (10 mM) repetitively triggered a similar transient Ca^2+^ increase. (C, D) Chloro (0.1 mM) induced a transient Ca^2+^ rise. Following the addition of the same concentration of caffeine, a small Ca^2+^ elevation was observed. These experiments demonstrate that chloro can induce Ca^2+^ increases and inhibit the following caffeine-triggered Ca^2+^ elevations. NS: *p* > 0.05.
(PDF)

S4 Fig Effect of 2-APB on caffeine-induced Ca^2+^ increases. (A) Caffeine (10 mM) repeatedly induced a similar Ca^2+^ increase, which was inhibited by the IP_3_R inhibitor 2-APB (B) ***: *p* < 0.001; NS: *p* > 0.05. This result indicates that caffeine-induced Ca^2+^ elevations arise partially from IP_3_R-mediated Ca^2+^ release.
(PDF)

S5 Fig U73122 inhibits caffeine-induced Ca^2+^ increases. (A) Caffeine (10 mM)-induced Ca^2+^ increases were measured using fluo-4 AM and confocal microscope, which were inhibited by the PLC inhibitor U73122 (B) ***: *p* < 0.001; NS: *p* > 0.05. This result suggests that PLC mediates caffeine-induced Ca^2+^ elevations.
(PDF)

S6 Fig Ryanodine inhibits caffeine-induced Ca^2+^ increases. (A) Caffeine (10 mM) and ryanodine (30 μM) were used to open and block RyRs, respectively, which induced Ca^2+^ increases measured with fluo-4 AM and confocal microscope. Following washout, caffeine-induced Ca^2+^ increases were still observed. (B) The summary results from 10 cells. ***: *p* < 0.001; NS: *p* > 0.05. These data imply that caffeine can induce Ca^2+^ elevations via RyR-independent pathway.
(PDF)

S7 Fig Chloro-induced force changes are reproducible. (A) A typical experiment performed as described in Fig. 7A. (B) After washing out chloro and a 40 min rest period, an equivalent experiment was performed. (C) The summarized results. NS: *p* > 0.05. These data indicate that chloro can time-independently induce biphasic changes in force.
(PDF)

S8 Fig Pax induces contraction. (A) Following ACH-induced contraction reached a plateau, pax added and resulted in an additional contraction. (B) Summarized contractions from 4 rings. This result demonstrates that blockade of BKs results in contraction in ASM. **: *p* < 0.01; ***: *p* < 0.001.
(PDF)

## References

[1] Deshpande DA, Wang WC, McIlmoyle EL, Robinett KS, Schillinger RM, An SS, et al Bitter taste receptors on airway smooth muscle bronchodilate by localized calcium signaling and reverse obstruction. Nat Med. 2010; 16: 1299–1304. 10.1038/nm.2237 20972434 PMC3066567

[2] An SS, Robinett KS, Deshpande DA, Wang WC, Liggett SB. Reply to: Activation of BK channels may not be required for bitter tastant-induced bronchodilation. Nat Med. 2012; 18: 650–651. 10.1038/nm.2734 22561815 PMC3371264

[3] Zhang CH, Chen C, Lifshitz LM, Fogarty KE, Zhu MS, ZhuGe R. Activation of BK channels may not be required for bitter tastant-induced bronchodilation. Nat Med. 2012; 18: 648–650; author reply 650–641. 10.1038/nm.2733 22561814

[4] Zhang T, Luo XJ, Sai WB, Yu MF, Li WE, Ma YF, et al Non-selective cation channels mediate chloroquine-induced relaxation in precontracted mouse airway smooth muscle. PLoS One. 2014; 9: e101578 10.1371/journal.pone.0101578 24992312 PMC4081631

[5] Zhang CH, Lifshitz LM, Uy KF, Ikebe M, Fogarty KE, ZhuGe R. The cellular and molecular basis of bitter tastant-induced bronchodilation. PLoS Biol. 2013; 11: e1001501 10.1371/journal.pbio.1001501 23472053 PMC3589262

[6] Liu QH, Zheng YM, Korde AS, Yadav VR, Rathore R, Wess J, et al Membrane depolarization causes a direct activation of G protein-coupled receptors leading to local Ca^2+^ release in smooth muscle. Proc Natl Acad Sci U S A. 2009; 106: 11418–11423. 10.1073/pnas.0813307106 19549818 PMC2708720

[7] Lifshitz LM, Carmichael JD, Lai FA, Sorrentino V, Bellve K, Fogarty KE, et al Spatial organization of RYRs and BK channels underlying the activation of STOCs by Ca^2+^ sparks in airway myocytes. J Gen Physiol. 2011; 138: 195–209. 10.1085/jgp.201110626 21746845 PMC3149436

[8] Malysz J, Rovner ES, Petkov GV. Single-channel biophysical and pharmacological characterizations of native human large-conductance calcium-activated potassium channels in freshly isolated detrusor smooth muscle cells. Pflugers Arch. 2013; 465: 965–975. 10.1007/s00424-012-1214-8 23344746 PMC3659209

[9] Kawano R, Tsuji Y, Sato K, Osaki T, Kamiya K, Hirano M, et al Automated parallel recordings of topologically identified single ion channels. Sci Rep. 2013; 3: 1995 10.1038/srep01995 23771282 PMC3683667

[10] Tuo QR, Ma YF, Chen W, Luo XJ, Shen J, Guo D, et al Reactive oxygen species induce a Ca^2+^-spark increase in sensitized murine airway smooth muscle cells. Biochem Biophys Res Commun. 2013; 434: 498–502. 10.1016/j.bbrc.2013.03.102 23583396

[11] Sai WB, Yu MF, Wei MY, Lu Z, Zheng YM, Wang YX, et al Bitter tastants induce relaxation of rat thoracic aorta precontracted with high K^+^ . Clin Exp Pharmacol Physiol. 2014; 41: 301–308. 10.1111/1440-1681.12217 24552423

[12] Hou P, Zeng W, Gan G, Lv C, Guo X, Zhang Z, et al Inter-alpha/beta subunits coupling mediating pre-inactivation and augmented activation of BK_Ca_ (beta2). Sci Rep. 2013; 3: 1666 10.1038/srep01666 23588888 PMC3627188

[13] Abdullaev IF, Rudkouskaya A, Mongin AA, Kuo YH. Calcium-activated potassium channels BK and IK1 are functionally expressed in human gliomas but do not regulate cell proliferation. PLoS One. 2010; 5: e12304 10.1371/journal.pone.0012304 20808839 PMC2924897

[14] Salkoff L, Butler A, Ferreira G, Santi C, Wei A. High-conductance potassium channels of the SLO family. Nat Rev Neurosci. 2006; 7: 921–931. 17115074 10.1038/nrn1992

[15] Anwer K, Oberti C, Perez GJ, Perez-Reyes N, McDougall JK, Monga M, et al Calcium-activated K^+^ channels as modulators of human myometrial contractile activity. Am J Physiol. 1993; 265: C976–985. 8238323 10.1152/ajpcell.1993.265.4.C976

[16] Nelson MT, Cheng H, Rubart M, Santana LF, Bonev AD, Knot HJ, et al Relaxation of arterial smooth muscle by calcium sparks. Science. 1995; 270: 633–637. 7570021 10.1126/science.270.5236.633

[17] Semenov I, Wang B, Herlihy JT, Brenner R. BK channel beta1 subunits regulate airway contraction secondary to M2 muscarinic acetylcholine receptor mediated depolarization. J Physiol. 2014; 589: 1803–1817. 10.1113/jphysiol.2010.204347 PMC3099031 21300746

[18] Zhou XB, Wulfsen I, Lutz S, Utku E, Sausbier U, Ruth P, et al M2 muscarinic receptors induce airway smooth muscle activation via a dual, Gbetagamma-mediated inhibition of large conductance Ca^2+^-activated K^+^ channel activity. J Biol Chem. 2008; 283: 21036–21044. 10.1074/jbc.M800447200 18524769 PMC3258941

[19] Brenner R, Perez GJ, Bonev AD, Eckman DM, Kosek JC, Wiler SW, et al Vasoregulation by the beta1 subunit of the calcium-activated potassium channel. Nature. 2000; 407: 870–876. 11057658 10.1038/35038011

[20] Tan X, Sanderson MJ. Bitter tasting compounds dilate airways by inhibiting airway smooth muscle calcium oscillations and calcium sensitivity. Br J Pharmacol. 2014; 171: 646–662. 10.1111/bph.12460 24117140 PMC3969078

