# Supplementary material for: Involvement of Large-Conductance Ca2+-Activated K+ Channels in Chloroquine-Induced Force Alterations in Pre-Contracted Airway Smooth Muscle
Source: PLoS One. 2015 Mar 30;10(3):e0121566. doi: 10.1371/journal.pone.0121566 (PMC4378962; doi:10.1371/journal.pone.0121566)
Supplement: S1 Fig — (A) STOCs were recorded at 10 mV, which were gradually inhibited by chloro. (B) The dose-dependent inhibition of amplitude and frequency from 5 cells. * denotes p < 0.05 (versus control); ** denotes p < 0.01 (versus control). These data show that chloro dose-dependently inhibits STOCs. (PDF) [file pone.0121566.s001.pdf]

Figure S1

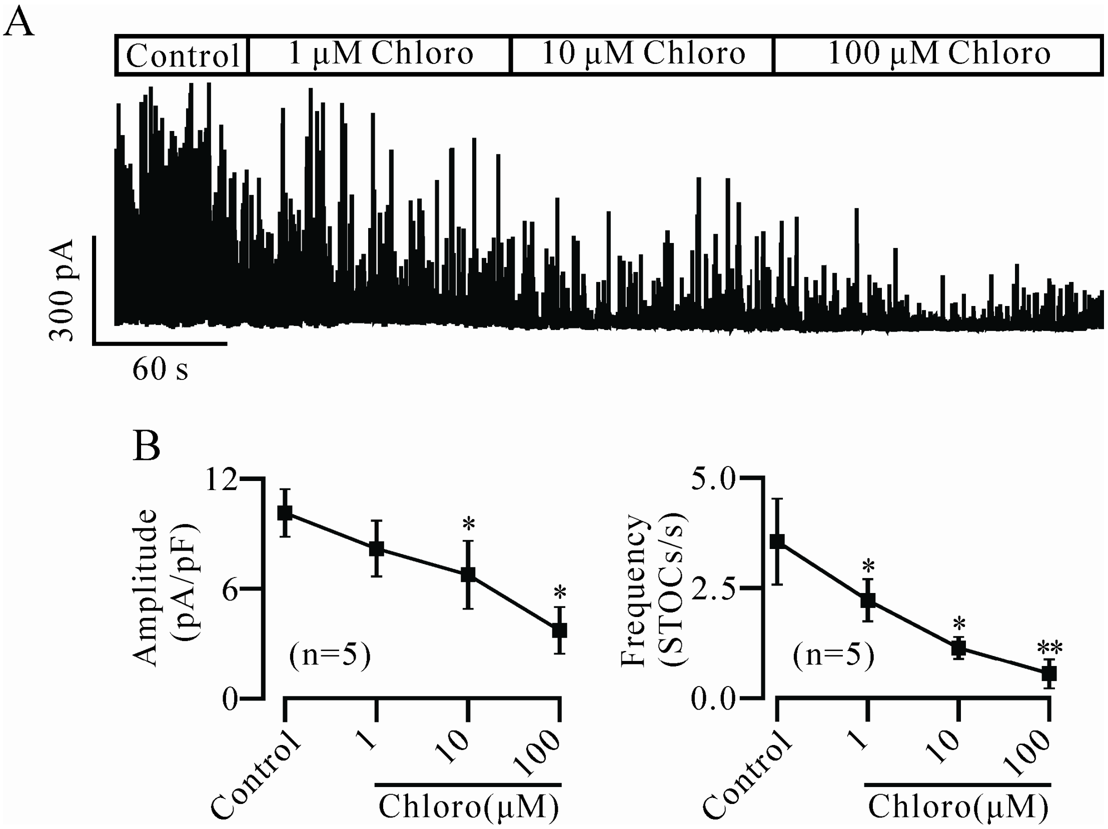

Figure S1. Chloro inhibits STOCs. (A) STOCs were recorded at 10 mV, which were gradually inhibited by chloro. **(B)** The dose-dependent inhibition of amplitude and frequency from 5 cells. * denotes $p < 0.05$ (versus control); ** denotes $p < 0.01$ (versus control). These data show that chloro dose-dependently inhibits STOCs.
