# Supplementary material for: Involvement of Large-Conductance Ca2+-Activated K+ Channels in Chloroquine-Induced Force Alterations in Pre-Contracted Airway Smooth Muscle
Source: PLoS One. 2015 Mar 30;10(3):e0121566. doi: 10.1371/journal.pone.0121566 (PMC4378962; doi:10.1371/journal.pone.0121566)
Supplement: S2 Fig — Ca2+ sparks from 8 cells were measured as described in Fig. 2A. (A) The frequency and (B) amplitude were markedly inhibited by 0.1 mM chloro. * denotes p < 0.05; *** denotes p < 0.001. These experiments demonstrate that low concentrations of chloro do not completely block, but rather inhibit RyRs. (PDF) [file pone.0121566.s002.pdf]

Figure S2

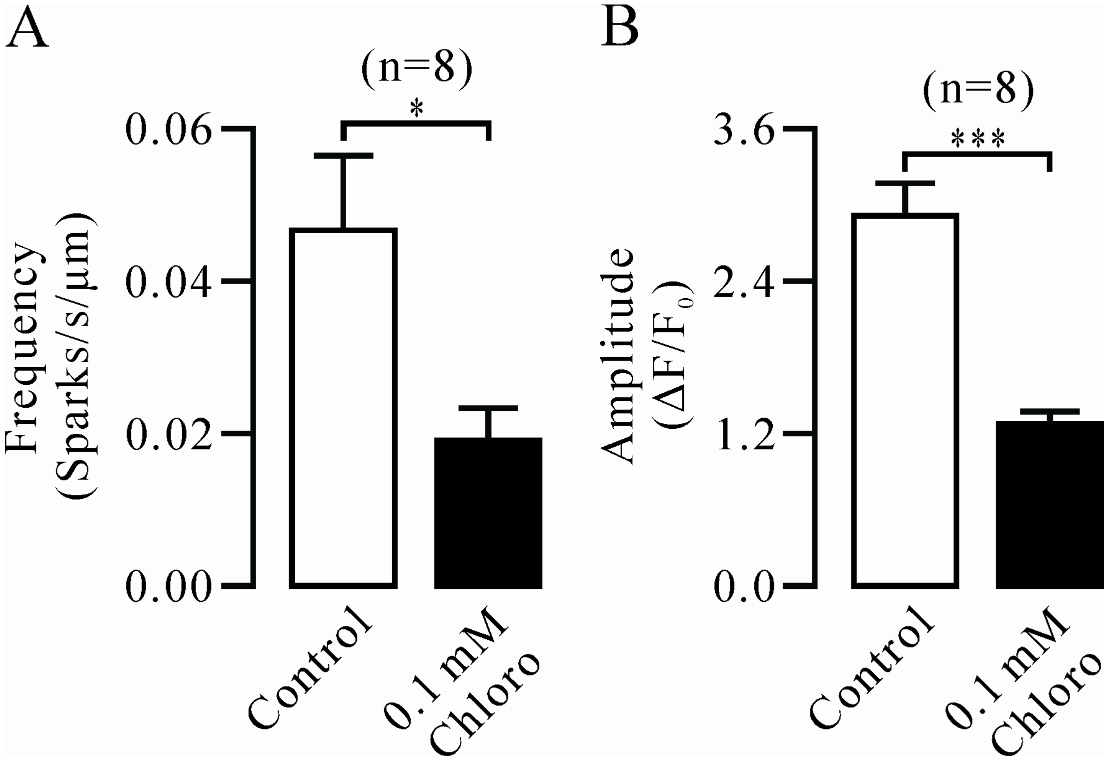

Figure S2. Chloro inhibits Ca^{2+} sparks. Ca^{2+} sparks from 8 cells were measured as described in Figure 2A. **(A)** The frequency and **(B)** amplitude were markedly inhibited by 0.1 mM chloro. * denotes $p < 0.05$; *** denotes $p < 0.001$. These experiments demonstrate that low concentrations of chloro do not completely block, but rather inhibit RyRs.
