# Supplementary material for: Involvement of Large-Conductance Ca2+-Activated K+ Channels in Chloroquine-Induced Force Alterations in Pre-Contracted Airway Smooth Muscle
Source: PLoS One. 2015 Mar 30;10(3):e0121566. doi: 10.1371/journal.pone.0121566 (PMC4378962; doi:10.1371/journal.pone.0121566)
Supplement: S3 Fig — (A, B) Caffeine (10 mM) repetitively triggered a similar transient Ca2+ increase. (C, D) Chloro (0.1 mM) induced a transient Ca2+ rise. Following the addition of the same concentration of caffeine, a small Ca2+ elevation was observed. These experiments demonstrate that chloro can induce Ca2+ increases and inhibit the following caffeine-triggered Ca2+ elevations. NS: p > 0.05. (PDF) [file pone.0121566.s003.pdf]

Figure S3.

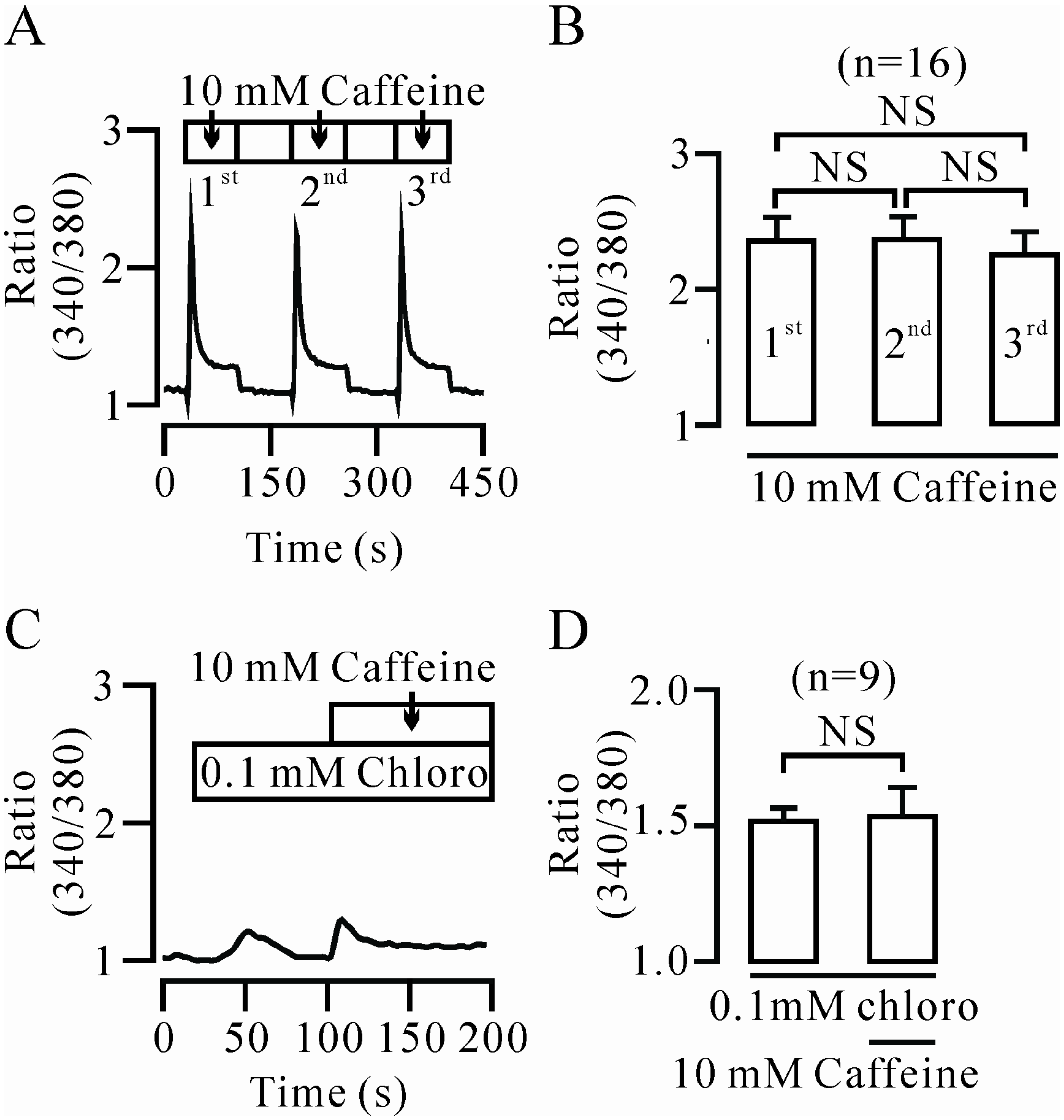

Figure S3. Effect of chloro and caffeine on intracellular Ca^{2+} levels. (A, B) Caffeine (10 mM) repetitively triggered a similar transient Ca^{2+} increase. **(C, D)** Chloro (0.1 mM) induced a transient Ca^{2+} rise. Following the addition of the same concentration of caffeine, a small Ca^{2+} elevation was observed. These experiments demonstrate that chloro can induce Ca^{2+} increases and inhibit the following caffeine-triggered Ca^{2+} elevations. NS: $p > 0.05$.
