# Supplementary material for: Involvement of Large-Conductance Ca2+-Activated K+ Channels in Chloroquine-Induced Force Alterations in Pre-Contracted Airway Smooth Muscle
Source: PLoS One. 2015 Mar 30;10(3):e0121566. doi: 10.1371/journal.pone.0121566 (PMC4378962; doi:10.1371/journal.pone.0121566)
Supplement: S4 Fig — (A) Caffeine (10 mM) repeatedly induced a similar Ca2+ increase, which was inhibited by the IP3R inhibitor 2-APB (B) ***: p < 0.001; NS: p > 0.05. This result indicates that caffeine-induced Ca2+ elevations arise partially from IP3R-mediated Ca2+ release. (PDF) [file pone.0121566.s004.pdf]

Figure S4

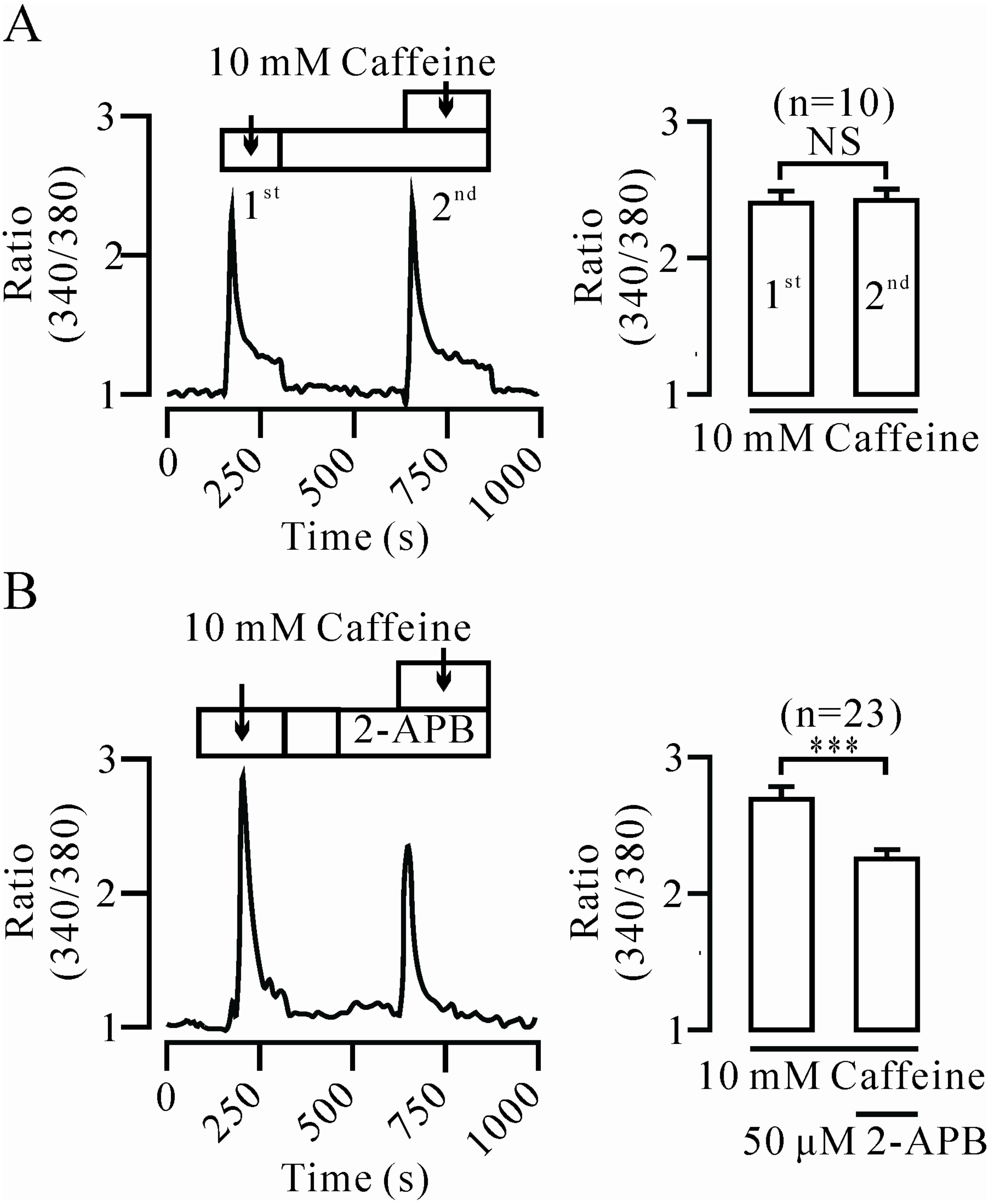

Figure S4. Effect of 2-APB on caffeine-induced Ca^{2+} increases. (A) Caffeine (10 mM) repeatedly induced a similar Ca^{2+} increase, which was inhibited by the IP_3R inhibitor 2-APB **(B)** ***: p < 0.001; NS: p > 0.05. This result indicates that caffeine-induced Ca^{2+} elevation arises partially from IP_3R-mediated Ca^{2+} release.
