# Supplementary material for: Involvement of Large-Conductance Ca2+-Activated K+ Channels in Chloroquine-Induced Force Alterations in Pre-Contracted Airway Smooth Muscle
Source: PLoS One. 2015 Mar 30;10(3):e0121566. doi: 10.1371/journal.pone.0121566 (PMC4378962; doi:10.1371/journal.pone.0121566)
Supplement: S5 Fig — (A) Caffeine (10 mM)-induced Ca2+ increases were measured using fluo-4 AM and confocal microscope, which were inhibited by the PLC inhibitor U73122 (B) ***: p < 0.001; NS: p > 0.05. This result suggests that PLC mediates caffeine-induced Ca2+ elevations. (PDF) [file pone.0121566.s005.pdf]

Figure S5

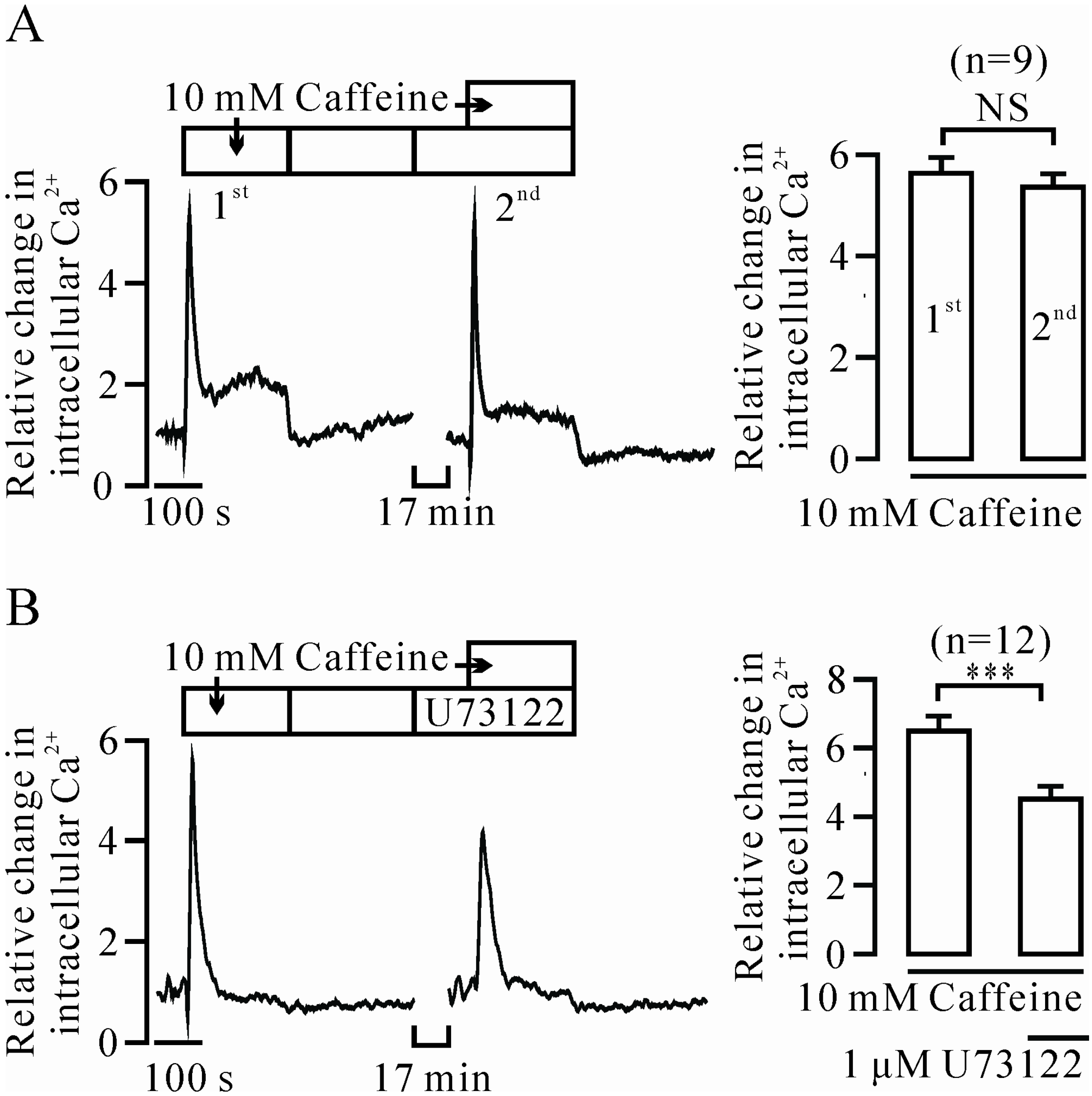

Figure S5. U73122 inhibits caffeine-induced Ca^{2+} increases. (A) Caffeine (10 mM)-induced Ca^{2+} increases were measured using fluo-4 AM and confocal microscope, which were inhibited by the PLC inhibitor U73122 **(B)** ***: $p < 0.001$; NS: $p > 0.05$. This result suggests that PLC mediates caffeine-induced Ca^{2+} elevations.
