# Supplementary material for: Involvement of Large-Conductance Ca2+-Activated K+ Channels in Chloroquine-Induced Force Alterations in Pre-Contracted Airway Smooth Muscle
Source: PLoS One. 2015 Mar 30;10(3):e0121566. doi: 10.1371/journal.pone.0121566 (PMC4378962; doi:10.1371/journal.pone.0121566)
Supplement: S6 Fig — (A) Caffeine (10 mM) and ryanodine (30 μM) were used to open and block RyRs, respectively, which induced Ca2+ increases measured with fluo-4 AM and confocal microscope. Following washout, caffeine-induced Ca2+ increases were still observed. (B) The summary results from 10 cells. ***: p < 0.001; NS: p > 0.05. These data imply that caffeine can induce Ca2+ elevations via RyR-independent pathway. (PDF) [file pone.0121566.s006.pdf]

Figure S6

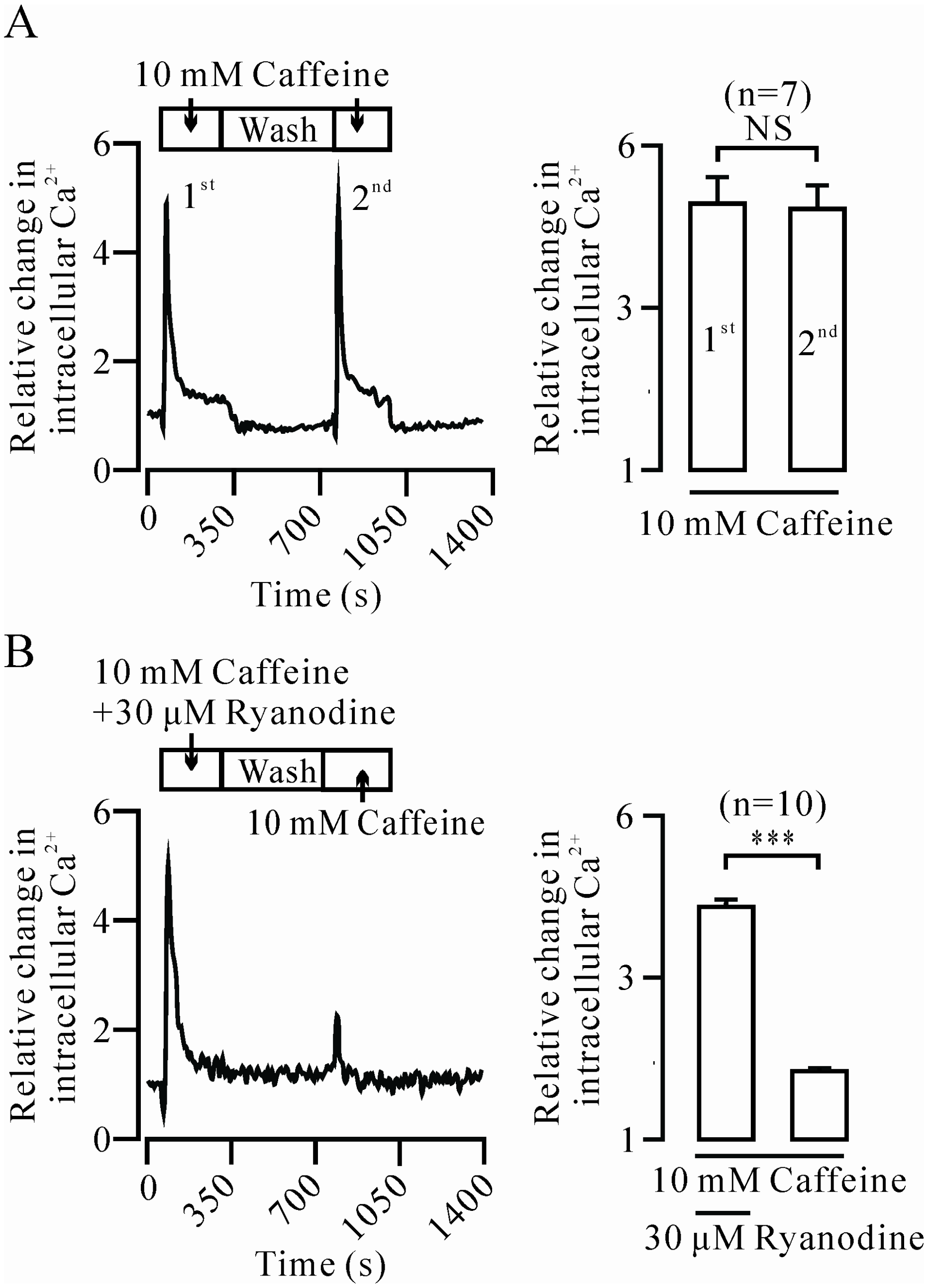

Figure S6. Ryanodine inhibits caffeine-induced Ca^{2+} increases. (A) Caffeine (10 mM) and ryanodine (30 μM) were used to open and block RyRs, respectively, which induced Ca^{2+} increases measured with fluo-4 AM and confocal microscope. Following washout, caffeine-induced Ca^{2+} increases were still observed. **(B)** The summary results from 10 cells. ***: $p < 0.001$; NS: $p > 0.05$. These data imply that caffeine can induce Ca^{2+} elevations via RyR-independent pathway.
