# Supplementary material for: Involvement of Large-Conductance Ca2+-Activated K+ Channels in Chloroquine-Induced Force Alterations in Pre-Contracted Airway Smooth Muscle
Source: PLoS One. 2015 Mar 30;10(3):e0121566. doi: 10.1371/journal.pone.0121566 (PMC4378962; doi:10.1371/journal.pone.0121566)
Supplement: S7 Fig — (A) A typical experiment performed as described in Fig. 7A. (B) After washing out chloro and a 40 min rest period, an equivalent experiment was performed. (C) The summarized results. NS: p > 0.05. These data indicate that chloro can time-independently induce biphasic changes in force. (PDF) [file pone.0121566.s007.pdf]

Figure S7

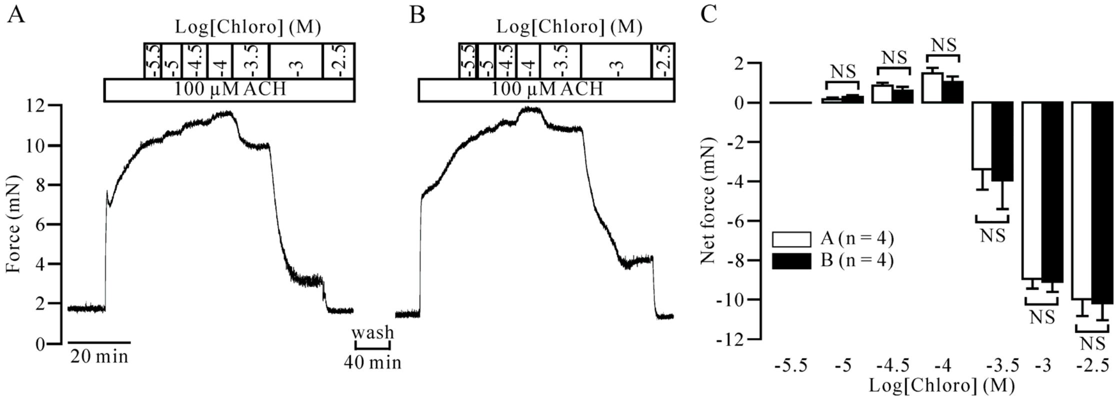

Figure S7. Chloro-induced force changes are reproducible. (A) A typical experiment performed as described in Figure 7A. **(B)** After washing out chloro and a 40 min rest period, an equivalent experiment was performed. **(C)** The summarized results. NS: $p > 0.05$. These data indicate that chloro can time-independently induce biphasic changes in force.
