# Supplementary material for: Involvement of Large-Conductance Ca2+-Activated K+ Channels in Chloroquine-Induced Force Alterations in Pre-Contracted Airway Smooth Muscle
Source: PLoS One. 2015 Mar 30;10(3):e0121566. doi: 10.1371/journal.pone.0121566 (PMC4378962; doi:10.1371/journal.pone.0121566)
Supplement: S8 Fig — (A) Following ACH-induced contraction reached a plateau, pax added and resulted in an additional contraction. (B) Summarized contractions from 4 rings. This result demonstrates that blockade of BKs results in contraction in ASM. **: p < 0.01; ***: p < 0.001. (PDF) [file pone.0121566.s008.pdf]

**Figure S8.**

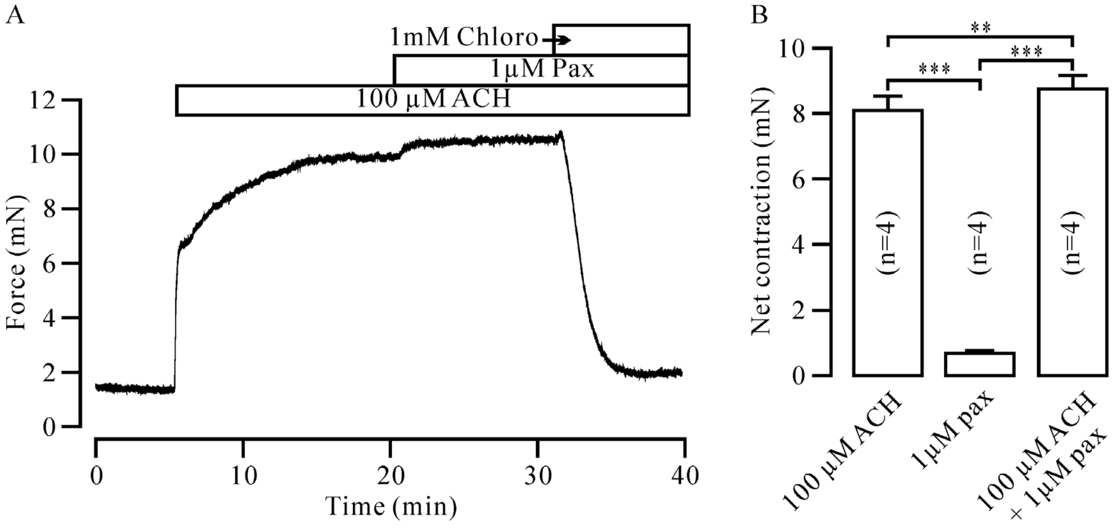


**Figure S8. Pax induces contraction. (A)** Following ACH-induced contraction reached a plateau, pax added and resulted in an additional contraction. **(B)** Summarized contractions from 4 rings. This result demonstrates that blockade of BKs results in contraction in ASM. **: $p < 0.01$; ***: $p < 0.001$.
